# Effects of changes in early retirement policies on labor force participation: the differential effects for vulnerable groups

**DOI:** 10.5271/sjweh.3946

**Published:** 2021-03-31

**Authors:** Karen M Oude Hengel, Carlos Riumallo-Herl, Jolinda LD Schram, D Nieboer, Allard J van der Beek, Alex Burdorf

**Affiliations:** Erasmus University Medical Center, Department of Public Health, Rotterdam, The Netherlands; Netherlands Organisation for Applied Scientific Research TNO, Department of Work Health Technology, Leiden, The Netherlands; Applied Economics, Erasmus University Rotterdam, Rotterdam, Netherlands; Department of Public and Occupational Health, Amsterdam UMC, VU University Amsterdam, Amsterdam Public Health Research Institute, Amsterdam, The Netherlands

**Keywords:** chronic disease, gender, older worker, regression discontinuity

## Abstract

**Objectives:**

This study investigated the effects of a national early retirement reform, which was implemented in 2006 and penalized early retirement, on paid employment and different exit pathways and examined whether these effects differ by gender, income level and health status.

**Methods:**

This study included all Dutch individuals in paid employment born six months before (control group) and six months after (intervention group) the cut-off date of the reform (1 January 1950) that fiscally penalized early retirement. A regression discontinuity design combined with restricted mean survival time analysis was applied to evaluate the effect of penalizing early retirement on labor force participation from age 60 until workers reached the retirement age of 65 years, while accounting for secular trends around the threshold.

**Results:**

The intervention group postponed early retirement by 7.41 months [95% confidence interval (CI) 6.11–8.72], and partly replaced this by remaining 4.87 months (95% CI 3.60–6.24) longer in paid employment. Workers born after the threshold, annually earning €25 000–40 000, spent 1.24 months (95% CI 0.31–2.18) more in economic inactivity than those born before. The working months lost to unemployment increased by 1.50 months (95% CI 0.30–2.71) for female workers and 1.99 months (95% CI 0.06–3.92) for workers reporting multiple chronic diseases.

**Conclusions:**

The national reform successfully prolonged working lives of older workers. However, workers with a middle income, female workers, and workers with chronic diseases were more vulnerable to premature exit from the labor market through unemployment or being without any income or benefit.

High and increasing youth unemployment rates in the 1970s and early 1980s (1) led to the implementation of an array of labor market policies to lower – or at least contain – these rates in most OECD countries. Policies included early retirement schemes for older workers that encouraged retirement before the statutory age (2). Early retirement schemes made it financially unattractive for older people to remain in the labor force, resulting in a drop in the average effective retirement age for male workers from 68.4 in 1970 to 61.5 years in the mid-1990s in Europe (3).

Since the late 1980s, it became apparent that the widespread use of early retirement schemes was no longer financially sustainable. In most OECD countries, these early retirement schemes were operated on a pay-as-you-go (PAYG) basis where the working population contributes to the benefits obtained by retirees (4). However, population ageing has expanded the fraction of occupationally inactive population (5) and, therefore, increased the overall dependency rate, which placed a great financial burden on a relatively smaller working population. This so-called old-age dependency ratio will continue to rise in OECD countries reaching 53.2% in 2050, up from 19.5% in 1975 (4). Consequently, reforming public pension systems has become a central policy issue across developed countries since the early 1990s.

This is also the case in The Netherlands, where the effective retirement age in the mid-1990s was even below the average retirement age in other OECD countries (61.0 versus 63.2 years, respectively) (3). In response to the financial concerns about the provision of pension income, the Dutch Government announced a reform in the summer of 2005 that would abolish the favorable tax deductions of contributions to early retirement schemes from 1 January 2006 onwards for workers born after 1 January 1950. While a pension reform was expected in the light of the pension debates in The Netherlands and other countries, the speed of the implementation and the sharp distinction between those born just before or after 1 January 1950 was unforeseen when the reform was announced.

Recent studies have concluded that the early retirement reform did prolong the working lives of Dutch workers, and this effect was stronger for males (6, 7). Using a similar basic dataset and the same policy measure as in the current paper, Boot et al (6) showed that a larger proportion of workers born in 1950 left paid employment through unemployment benefits. Our study contributes to the literature by building on existing studies and provides a more thorough and nuanced methodological evaluation of the reform. First, we combine a sharp regression discontinuity (RD) design with restricted mean survival time (RMST) analysis to estimate the causal effect of the policy on the time in months spent on the labor rather than changes in proportions in different work states. In this sense, the reform acted as ‘a natural experiment’ with a clear cut-off of the treatment assignment and provides estimates that are the closest to a randomized controlled trial in observational data (8). The combination with time analysis is rather scarce, and the current study serves as an example for future studies using panel data. This leads not only to a methodological improvement but provides us with more nuanced policy-relevant results by presenting a precise measure of the additional number of months spent in paid employment encouraged by the reform.

Second, we provide a more substantive contribution by exploring the heterogeneous effects of the reform on labor force participation by groups of workers categorized by gender, socioeconomic status and health. While previous studies have evaluated the impact of this reform by gender, they have ignored the other dimensions that have been reported as important determinants of early retirement (9, 10). For example, workers with poor health have an increased risk to leave paid employment through early retirement (11, 12). As early retirement, disability and unemployment benefits might act as communicating vessels to exit paid employment (13), it could be hypothesized that vulnerable workers choose or are forced to leave paid employment through other pathways with reduced social benefits when the pathway of early retirement becomes less attractive. Thus, exploring effect heterogeneity also among socioeconomic status and health is of great policy relevance.

Against this background, the objectives of this study were to (i) investigate the effects of the reform on duration of paid employment and the timing to other pathways out of paid employment and (ii) examine whether these effects differed across groups of workers.

## Methods

### Dutch pension system and reform

The Dutch pension system consists of three pillars. The first pillar is the old-age pension, which is the basic old age pension provided by the government to all Dutch residents when they reach the statutory retirement age. The second pillar comprises supplementary collective pension schemes, which are linked to specific companies or industries and most employees are entitled to benefits from this pillar. Early retirement schemes are determined by negotiations between labor unions and employer organizations at the sectoral level. Participation in these collective schemes is mandatory for each individual employee, ensuring coverage by the sector pension, which allows workers to retire earlier. As contributions to the sectoral pensions were tax deductible, early retirement was the social norm among Dutch workers before the policy reform. On average, the first and second pillar both make up half of the pension entitlements (14). The third pillar consists of individual voluntarily built-up savings supplementary to the public and sector pensions.

The reform in this study relates to the second pillar of the Dutch pension system. Since 1 January 2006, early retirement schemes were integrated into the capital funded occupational pension system. As a consequence, early retirement benefits – which could be applied for from the age of 60 – were reduced to discourage early retirement. To exemplify how much the new system penalized early retirement compare a person born before 1950, who could retire at the age of 62 years and three months with 70% of their gross wage, to a worker born in 1950 or later who would have to work an additional 13 months to retire with the same percentage of gross wage or retire at 62 years and three months with only 64% of their gross wage as retirement income: a 6 percentage point drop in retirement income (7). This penalty applied for any individual born after 1950 who retired between the ages of 60 and 65 – the full legal retirement age.

### Datasets and study population

Register data of Statistics Netherlands were used, covering the entire Dutch population. The study sample consisted of individuals born six months before (control group) and six months after (intervention group) 1 January 1950. Individuals at the age of 60 years were included in the study because early retirement is possible from this age onwards. Of the entire population, 49.0% of the control group and 51.2% of the intervention group were working at baseline and thus included in our study.

These data were enriched with data on dispensed medicines and personal gross income from additional register data of Statistics Netherlands. Additionally, monthly information was included on main income components, social benefit pensions and gross wages, derived from the Dutch tax registers and stored in the social statistical database (SSB) (15). Individuals were followed up until the age of 65, the Dutch statutory retirement age at the time. Finally, information regarding whether and when a participant died during follow-up was also included. The final study sample consisted of 102 617 participants (49 501 and 53 116 in the control and intervention groups, respectively).

### Employment status

Monthly employment status was defined based on the most important source of income and categorized into paid employment, disability benefits, unemployment benefits, early retirement benefits, and economically inactive. Employed individuals were defined as having their main source of income from paid employment. Self-employment was not included in the current study as the regulations regarding social benefits differ substantially from those in paid employment. Early-retired persons were defined as those who received a company pension as their main source of income but had not reached the Dutch statutory retirement age yet. Unemployed persons received either unemployment benefits due to having lost their job or social security benefits. Receiving disability benefits was defined as when disability benefits represented ≥50% of total monthly income. Finally, economically inactive persons did not have personal income or benefits because they stopped for reasons such as being a homemaker or retired without receiving early retirement benefits. If an individual reached a specific exit pathway for ≥3 months, then this pathway was considered an actual event. Only the first event over time was considered in this study. Additionally, whether and when a participant died was added as a competing event.

### Chronic diseases

The presence of a chronic disease at baseline was based on the database of dispensed medicines in 2006 from Statistics Netherlands, which contains information on purchased drugs that were reimbursed by the health care insurances. Dispensed medicines are classified into anatomic, therapeutic, chemical (ATC) classification codes, according to the WHO (World Health Organization) drug classification system of 2010 (16). Previous research has used these ATC codes to identify specific chronic diseases and estimate the prevalence among the working population (17, 18). Following these definitions, six chronic diseases were identified: cardiovascular diseases, respiratory diseases, inflammatory diseases, diabetes, mental disorders (depressive, anxiety and sleep disorders) and psychotic disorders. From this, workers were categorized into having 0, 1 or ≥2 chronic diseases.

### Income level

Income level was based on the database of annual personal gross income of Statistics Netherlands. Since retirement decisions rely on personal income and savings, we use personal gross income at the age of 54 as a measure of economic wellbeing. Hence, annual personal gross income was measured five years before the reform and, thus, the reform is less likely to have influenced income: 2003 for those born in 1949 (control group) and 2004 for those born in 1950 (intervention group). Income was adjusted for inflation between both birth cohorts and then divided into four categories: <€25 000, €25 000–40 000, €40 000–55 000, and >€55 000.

### Statistical analyses

To evaluate the effect of disincentivizing early retirement a RD approach with a RMST design was used to estimate the causal effect of penalizing early retirement on labor force participation between 60–65 years (19). Under this approach, two populations are compared where the assignment to treatment depends only on whether an individual observed assignment variable (ie, running variable) exceeds a cut-off point. In the current study, the intervention group – those who lose the tax incentives for early retirement – was defined as those who are born just on or after 1 January 1950. The benefits of using an RD approach is threefold. First, one can use the observed data to verify whether the assumption of exogeneity is likely to hold. This is explained in the following paragraph. Second, RD takes advantage of a natural experiment setting whereby the variation isolated by the cut-off can be considered as good as random due to the inability of individuals in the study to influence the treatment allocation (20). Finally, it represents a transparent and understandable approach to estimate the causal effects of a policy measure.

The intuition behind this RD approach is that the treatment is exogenous in the vicinity around the threshold and, thus, offers an estimation of the causal effect of the policy. Two assumptions are important to verify. First, individuals should not be able to manipulate the assignment variable to treatment (8), which is the case in our study by using date of birth. The second condition is that individuals on either side of the age threshold introduced by the reform are statistically comparable (8). To verify this, we show graphically that individuals around the threshold are statistically the same while account for potential secular trends. The combination of both conditions entails that for individuals in the vicinity of the threshold there is only variation in the treatment status that is uncorrelated with any baseline characteristics.

The statistical analyses consisted of three steps. In the first step, monthly employment records from age 60 until 65 were used to calculate the observed number of months (up to 60 months) that each worker spent in paid employment before exiting the labor force. Then for each individual, we identified the specific exit route for that individual (ie, disability benefits, unemployment benefits, early retirement, economic inactivity or death). The monthly employment records were also used to identify censored individuals. Workers were censored at 60 months if they were still working at the age of 65 (ie, statutory retirement age) or at their latest observation in paid employment when they (i) left paid employment through any of the pathways other than those of interest, (ii) became self-employed, or (iii) were missing (eg, due to emigration). A descriptive figure of the Kaplan-Meier cumulative incidence curves for each specific exit route is constructed based on this data.

In the second step, the monthly employment records were used to estimate the RMST in number of months for each specific exit pathway, which was the outcome in our RD equations (21). In practice, this was done using the approach by Andersen et al (22) where the cause-specific RMST is calculated with a “leave-one-out” technique in combination with Kaplan-Meier survival curves starting at the age of 60 and until the age of 65. This approach enables us to account for right-hand censoring in each of the exit pathways. The advantages of this RMST approach are the ease in interpretation of the coefficients (ie, actual months) and the possibility to include the RMST estimates in other statistical models.

In the final step, the estimated RMST for each individual was included as the outcome in a RD analysis by comparing the differences in the mean employment time and time in the different pathways between the intervention group and control group for each exit route separately, while accounting for secular trends around the threshold. All RD models were controlled for covariates (ie, gender, income level, chronic disease). For a full description of the RD model, refer to the supplementary material (www.sjweh.fi/show_abstract.php?abstract_id=3946, mathematical model). As death was not considered a primary outcome, the effect of the reform on death is not further presented in this paper.

To evaluate heterogeneity, we estimated the equation above stratified by gender, income level, and the presence of a chronic disease. To verify the robustness of the results, sensitivity analyses were conducted using quadratic functions in both sides of the threshold. In the main analysis, a bandwidth of six months around the threshold was used, and bandwidths of three and twelve months were used in the sensitivity analyses. The trade-off is such that, as the bandwidth is increased, it is likely that individuals in the treatment and control group are less balanced, thus, reducing the internal validity. However, with a smaller bandwidth, the sample size decreases and statistical power will be reduced.

Statistical analyses were performed in RStudio statistical software, version 1.1.463, using Rdrobust package for the RD estimation.

## Results

Table 1 presents the characteristics of the total study population (N=102 617), 49 501 workers (61.2% male) constituted the control group, while 53 116 workers (61.1% male) composed the intervention group. Approximately half of the respondents had ≥1 chronic disease (49.9% and 49.7% in the control and intervention groups, respectively). Baseline characteristics, as required for the RD approach, were similar between the intervention and control groups. Supplementary figure S1 shows the graphical balance in baseline characteristics for those in the treatment and control group. When stratified for gender, income level or chronic disease, slightly more female workers in the intervention group were in the €40 000–55 000 income bracket compared to the control group [β 0.09 (95% CI 0.01–0.17)].

**Table 1:** Descriptive statistics at baseline and labor force exit.

	Control group N=49 501		Intervention group N=53 116	
	
	N	%	N	%
Gender (male)	30 305	61.2	32 434	61.1
Income (€)				
≤25 000	12 860	26.0	13 478	25.4
25 000–40 000	12 351	25.0	12 378	23.3
40 000–55 000	11 361	23.0	12 529	23.6
≥55 000	12 929	26.1	14 731	27.7
Chronic disease				
0	24 820	50.1	26 703	50.3
1	15 929	32.2	17 113	32.2
≥2	8752	17.7	9300	17.5
Specific disease				
Cardiovascular	11 869	24.0	12 275	23.1
Inflammatory	10 972	22.2	11 958	22.5
Psychological	6980	14.1	7528	14.2
Respiratory	3376	6.8	3736	7.0
Diabetes	2336	4.7	2402	4.5
Psychotic	214	0.4	221	0.4
Labor force exit				
Early retirement	24 316	49.8	15 375	29.4
Disability benefits	1327	2.7	1854	3.5
Unemployment benefits	3510	7.2	5697	10.9
Economically inactive	2749	5.6	3126	6.0

**Figure 1:**
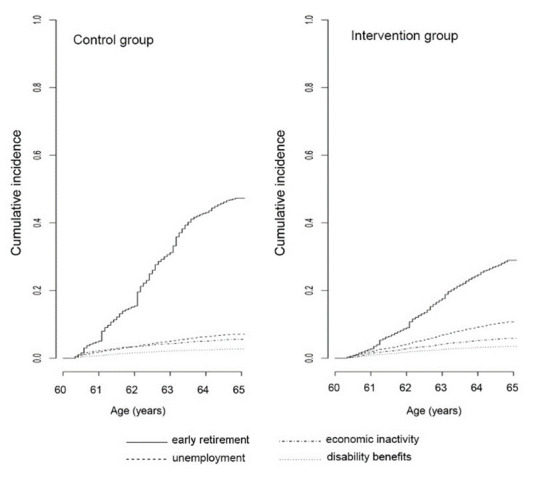
Cumulative incidence curves of early exit from paid employment through the four specific pathways in the control and intervention groups.

**Figure 2:**
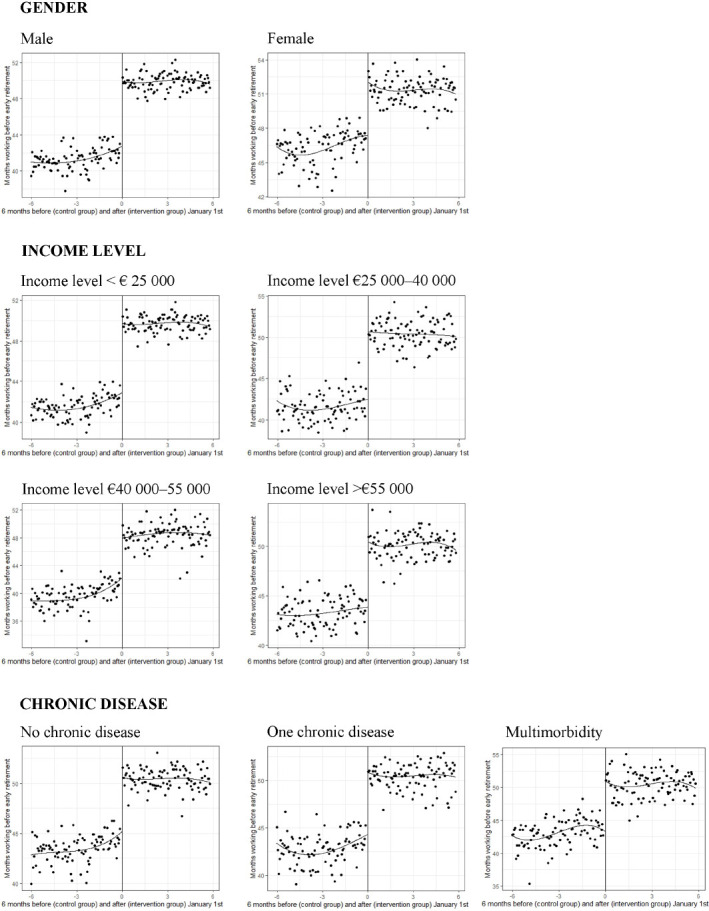
Stratified regression discontinuity figures for workers born 6 months before or after the cut-off of January 1st 1950 for the time workers were in paid employment before they exit through early retirement, stratified for gender, income level, and chronic disease. Each scatter plot represents the average outcome for individuals grouped into 100 bins at each side of the threshold.

Figure 1 shows a lower probability to exit from paid employment through early retirement in the intervention compared to control group, while the probability to exit from paid employment through other pathways was slightly higher for the intervention compared to control group (absolute probabilities for each pathway are presented in supplementary table S1). Almost half of the workers born in 1949 (49.8%) left paid employment through early retirement compared to less than one third of the workers born after the policy threshold (29.4% as shown in table 1). Figure 2 shows the effect heterogeneity of reform eligibility on the proportion of workers who exit paid employment through early retirement by gender, income and health. This figure shows important discontinuities but also differences in magnitudes for each group.

Table 2 shows that working months lost due to early retirement decreased by 7.41 months (95% CI 6.11– 8.72) in the intervention compared to control group, coinciding with prolonged paid employment by 4.87 months (95% CI 3.60–6.24). Furthermore, workers in the intervention group left paid employment through other pathways more often than the control group, but this was not statistically significant for any specific pathway. With regard to gender, male workers in the intervention group postponed early retirement by 8.50 months (95% CI 6.81–10.23) compared to the control group and replaced this by spending additional months in paid employment (6.32 months (95% CI 3.79–8.84). After the reform, women postponed early retirement by 5.63 months (95% CI 3.65–7.60). They replaced this by more months in unemployment (1.50 months, 95% CI 0.30–2.71) but also by spending additional months in paid employment (2.09 months, 95% CI -0.09–4.29), albeit the latter was not statistically significant.

**Table 2:** Regression discontinuity (RD) estimates of working months and months in different exit pathways from paid employment comparing the intervention to the control group as reference ^a^. Significant results (P-value <0.05) are presented **in bold**.

	Addition months spent in paid employment		Working months lost due to early exit through ^b^							

			Early retirement		Disability benefits		Unemployment		Economic inactivity	
				
	Months	95% CI	Months	95% CI	Months	95% CI	Months	95% CI	Months	95% CI
All ^c^	**4.87**	**3.60–6.24**	**-7.41**	**-8.72– -6.11**	0.47	-0.07–1.01	0.61	-0.23–1.44	0.51	-0.20–1.21
Gender^d^										
Female	2.09	-0.09–4.26	**-5.63**	**-7.60– -3.65**	0.28	-0.57–1.13	**1.50**	**0.30–2.71**	0.74	-0.77–2.24
Male	**6.75**	**5.00–8.51**	**-8.51**	**-10.23– -6.80**	0.52	-0.19–1.23	0.26	-1.23–1.06	0.32	-0.23–0.87
Income (€)^e^										
≤25 000	**3.35**	**0.58–6.12**	**-5.82**	**-8.20– -3.45**	0.26	-0.84–1.36	0.32	-1.35–1.99	-0.05	-2.25–2.15
25 000–40 000	**5.07**	**2.24–7.90**	**-8.08**	**-10.86– -5.30**	1.19	-0.07–2.43	0.15	-1.68–1.98	**1.24**	**0.31–2.18**
40 000–55 000	**4.41**	**1.59–7.23**	**-7.09**	**-9.87– -4.32**	0.99	-0.21–2.19	0.94	-0.82–2.70	0.45	-0.34–1.23
≥55 000	**6.32**	**3.79–8.84**	**-7.98**	**-10.38– -5.59**	-0.37	-1.17–0.44	1.04	-0.42–2.50	0.22	-0.66–1.10
Chronic disease^f^										
No	**5.70**	**3.76–7.65**	**-7.02**	**-8.85– -5.20**	0.23	-0.88–0.42	-0.30	-1.48–0.88	0.75	-0.19–1.69
One	**3.45**	**1.02–5.88**	**-6.86**	**-9.19– -4.53**	0.86	-0.14–1.86	1.29	-0.23–2.81	0.38	-0.89–1.65
Multiple	**4.99**	**1.77–8.20**	**-9.12**	**-12.14– -6.10**	1.85	-0.17–3.54	**1.99**	**0.06–3.92**	0.30	-2.06–1.47

a RD robust design with a bandwidth of 6 months (h=6) and a cubical polynomial (p=3).

b The sum of time in early retirement does not necessarily equal the time spent in other activities because each model is estimated independently and we have not included death as one of the pathways.

c Models were corrected for gender, personal gross income and chronic disease.

d Models were corrected for personal gross income and chronic disease.

e Models were corrected for gender and chronic disease.

f Models were corrected for gender and personal gross income.

The reform significantly delayed early retirement for all income levels, ranging from -5.82 months (95% CI -8.20– -3.45) for the lowest income level up to -8.08 months (95% CI -10.86– -5.30) for workers annually earning €25 000–40 000 (table 2). Workers from all income levels replaced this largely by additional months in paid employment, with the highest gain for workers with the highest income [6.32 months (95% CI 3.79–8.84)]. Even though workers in all income groups left paid employment earlier through other pathways, this was only significant for workers with an income of €25 000–€40 000, as they lost 1.24 working months (95% CI 0.31–2.18) through economic inactivity.

Additionally, the reform postponed early retirement among workers with one chronic disease by 6.86 months (95% CI 4.53–9.19), while this increased to 9.12 months (95% CI 6.10–12.14) for workers with multiple chronic diseases (table 2). This postponement in early retirement was partly replaced by prolonging paid employment (3.45 months (95% CI 1.02–5.88) for workers with one chronic disease and 4.99 months (95% CI 1.77–8.20) for workers with multiple chronic diseases). Both, workers with 1 and ≥2 chronic diseases in the intervention group lost more working months through disability and unemployment benefits than their counterparts in the control group. This was, however, only significant for workers with multimorbidity in the intervention compared to control group, as they lost 1.99 months (95% CI 0.06–3.92) more due to unemployment benefits.

In general, sensitivity analyses conducted with varying bandwidths and with a quadratic polynomial form showed similar results (supplementary tables S2–4).

## Discussion

This study shows that the Dutch policy reform was effective in delaying exit from paid employment through early retirement and prolonging paid employment among workers, in line with previous research (6, 23). However, differences were found across groups of workers. Workers with the highest income level and male workers in the intervention group fulfilled this postponement largely by remaining in paid employment compared to their counterparts in the control group. Workers annually earning €25 000–40 000 spent more months in economic inactivity and female workers spent more months in unemployment. Additionally, workers with chronic diseases in the intervention group were only partly able to replace their postponement with prolonging paid employment than workers in the control group, resulting in the fact that workers in the intervention group lost more paid employment months due to disability and unemployment benefits.

The reform aimed to discourage early retirement by disincentivizing rather than forcing workers to prolong their working careers. This was reflected by the fact that both women and men postponed early retirement, by 5.63 and 8.51 months respectively, which is below the 13 months required to receive the same amount of benefits as people born before 1950. In line with a previous study (6), female workers born from 1950 onwards replaced the postponement of early retirement by spending more months in unemployment and – to a lesser extent – economic inactivity, while corresponding male workers spending more months in paid employment. This difference in effect on the reform between gender needs to be explained by the different labor market attachment across genders in The Netherlands. Females mainly worked part-time, which was also reflected by the fact that almost 80% of the female workers were in the lowest two income levels. They have probably a lower need to prolong their working careers than men.

With regard to income level, the largest impact of the reform was found among workers with the highest income who replaced early retirement with paid employment, while workers with lower incomes exited paid employment more often through other pathways. The latter is undesirable because receiving disability benefits or becoming unemployed might have negative health consequences for individuals (24). In light of interpreting the effect of the reform, it needs to be highlighted that workers with a higher income level – and probably more savings – could also counter the economic consequences induced by disincentivizing early retirement more easily (7, 25). The Health Council of The Netherlands has recommended the government to monitor these socioeconomic (health) differences with respective to prolonging working careers (26).

Furthermore, workers with 1 or ≥2 chronic diseases postponed early retirement by 6.86 and 9.12 months, respectively. This is in line with a study of Pedersen et al (27) that showed a decline in disability pensions after a structural reform of the Danish Disability Pension Act. The interpretation of our effect is twofold. Previous studies showed that workers with multimorbidity (28) or poor health (29) had an increased risk to leave paid employment through early retirement, which could be seen as a health protection mechanism. When the reform discouraged exiting paid employment through early retirement, these workers left the labor force earlier through involuntary pathways (ie, disability benefits and unemployment benefits). Thus, early retirement and these social security pathways act as communicating vessels (13). On the other hand, workers with chronic diseases partly compensated the postponement of early retirement by prolonging their working lives. However, keeping workers with a chronic disease in paid employment longer could have negative consequences for their productivity and long-term health (30).

As many countries are currently discussing reforms in their pensions systems that penalize early retirement, policy-makers could benefit from the conclusions drawn in the current study. In particular, our results show that discouraging early retirement does lead to longer working lives. However, the study also showed that reforming the pension system could lead to effects in other social programs, emphasizing the interconnection among social benefits, which need to be paid by the society. Additionally, although such reforms can indeed delay retirement, the current study offers a word of caution for policy-makers. They need to be aware of the societal and individual side effects of these kind of reforms across different groups when implementing reforms prolonging working lives.

This study demonstrates that the RD design combined with survival data is a valuable approach to evaluate interventions with a strictly defined cut-off date. Taking the assumptions into account, the RD design can resemble a randomized controlled trial and estimate causal effects (31). As these methods are relatively new in the field of occupational health, researchers need to be encouraged to report the study quality and to promote transparency on the validity of the results by reporting five key elements (19): (i) a discussion of the RD validation conditions within the specific context, (ii) a clear presentation of the assignment rule, (iii) a test of the distribution of baseline covariates between the intervention and control group, (iv) a graph of the assignment variable to show no bunching of the data around the cut-off, and (v) several sensitivity analyses with varying bandwidth and function forms to check for robustness.

Major strengths of the current study include its large study population and administrative data on all variables. This enabled not only the in-depth evaluation of the effect for specific demographic subgroups of the population but also the ability to distinguish workers with chronic conditions from those free of disease. The current study, however, is not without limitations. First, as applying a RD design requires more statistical power, it was not possible to further stratify, for instance for both gender and income level. Second, the RD design estimates local average intervention effects and the results cannot be extended simply to other generations, eg, workers who were <50 years at the time of the reform. Third, we used register data on prescribed medication use to identify the five most common diseases. The register did not include over the counter medication such as painkillers that may be used for particular chronic diseases. Also, a chronic disease that requires treatment without medication was not included. Hence, we were unable to identify prevalent chronic diseases such as musculoskeletal disorders or less severe psychological health problems (eg, burnout) that are related to premature exit out of paid employment (32). Likewise, less common diseases, such as cancer, were also not included because the prevalence in the workforce was too low.

To conclude, the reform to discourage early retirement was effective in prolonging working lives. However, differences were found across groups of workers. Workers with the highest income and male workers largely replaced the postponement of early retirement by prolonging their working lives. Workers with a middle income lost more paid employment months through disability benefits, whereas female workers lost more paid employment months due to unemployment benefits. Workers reporting 1 or ≥2 chronic diseases postponed their early retirement but were unable to fulfill this entirely with paid employment.

## Supplementary material

Supplementary material
