# Supplementary material for: Effects of changes in early retirement policies on labor force participation: the differential effects for vulnerable groups
Source: Scand J Work Environ Health. 2021 Mar 31;47(3):224–32. doi: 10.5271/sjweh.3946 (PMC8126444; doi:10.5271/sjweh.3946)
Supplement: Supplementary material [file SJWEH-47-224-S001.pdf]

Effects of changes in early retirement policies on labor force participation: the differential effects for vulnerable groups[1]

by Karen M Oude Hengel, PhD,[2] C Riumallo-Herl, PhD, Jolinda LD Schram, MSc, D Nieboer, MSc, Allard J van der Beek, PhD, Alex Burdorf, PhD

1. *Supplementary material*
2. *Correspondence to: Karen Oude Hengel, PhD, Erasmus University Medical Center, Department of Public Health, PO Box 2040, 3000 CA Rotterdam, The Netherlands. [E-mail: k.oudehengel@erasmusmc.nl]*

Supplementary: Mathematical model

Separate regression discontinuity models were used to estimate the effects of the reform for each of the outcomes of interest (i.e. months in paid employment, exit paid employment through disability benefits, unemployment, or economic inactivity, and death). The equation presented in this supplementary file reflects the formula for the outcome time spent in employment.

The following equation was used to estimate the time spent in employment as the outcome of our regression discontinuity:

$$Y_i = f(R) + \beta * T_i + f'(R) * T_i$$

Where Y_i is the outcome of our study which corresponds to the number of months an individual remains employed after the age of 60; $f(R)$ represents a flexible polynomial function on date of birth for those born before January 1st 1950, this accounts for the secular trend on months worked below the threshold, T_i is an indicator of whether the individual is the treatment and control group, and $f'(R)$ is the polynomial function accounting for the secular trends for individuals born after January 1st, 1950. From this, β represents the coefficient of interest which reflects the average difference in months between those born before and after January 1st, 1950.

Supplementary figure S1. RD-plots of the proportion of population born 6 months before or after the cut-off of January 1st 1950 stratified for baseline characteristics

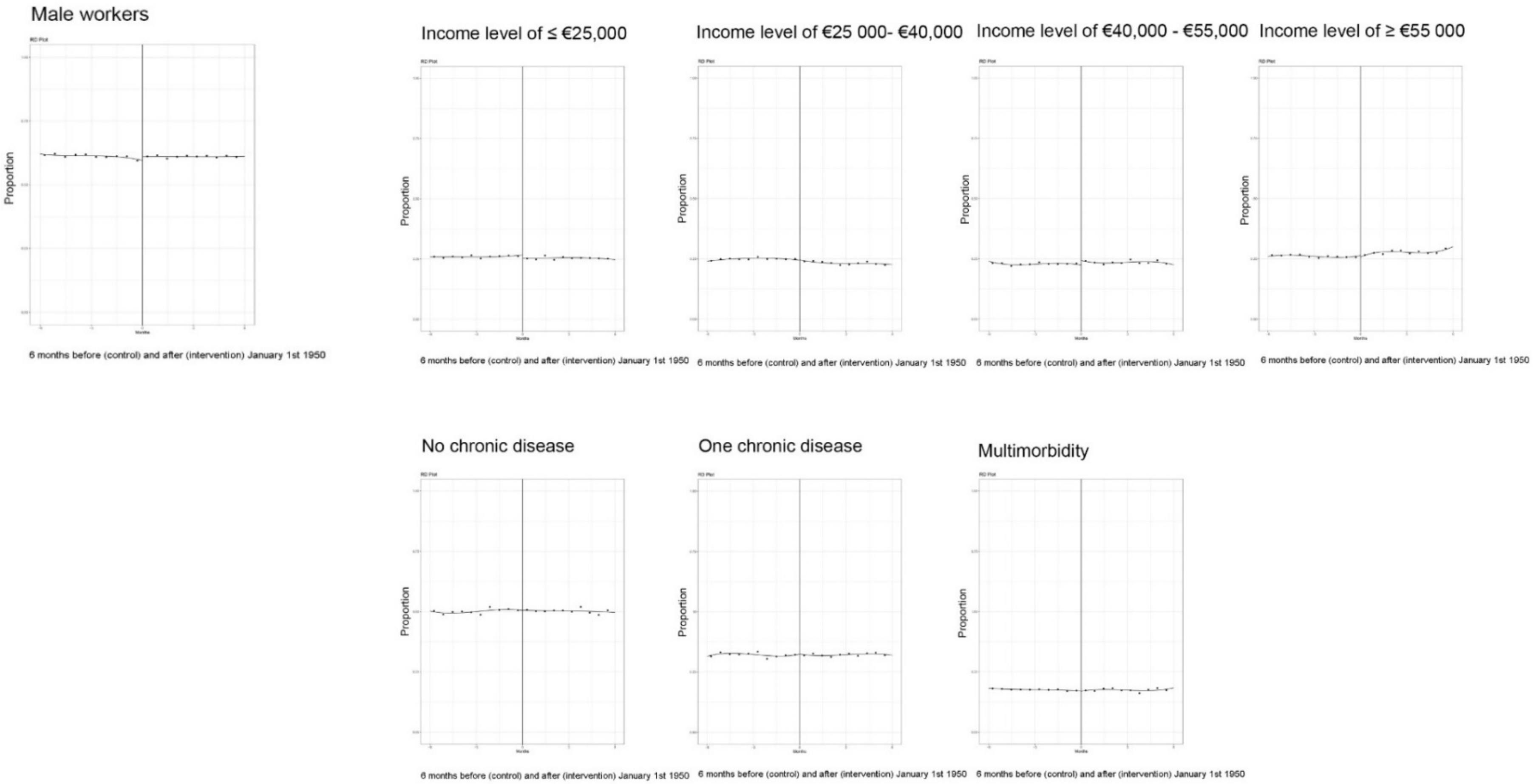

Supplementary table S1. Absolute probabilities to leave paid employment through any of the pathways

	Control group				Intervention group			
	**Exit from paid employment**				**Exit from paid employment**			
	Early retirement	Disability benefits	Unemployment benefits	Economic Inactivity	Early retirement	Disability benefits	Unemployment benefits	Economic Inactivity
All	47.3%	2.7%	7.1%	5.6%	28.9%	4.9%	10.7%	5.9%
Gender								
Female	46.8%	2.7%	7.2%	5.5%	28.9%	3.4%	11.0%	5.8%
Male	47.6%	2.7%	7.0%	5.6%	29.0%	3.6%	10.5%	5.9%
Income								
≤ €25 000	46.6%	2.7%	7.1%	5.6%	28.9%	3.4%	10.6%	5.9%
> €25 000 & ≤40 000	47.5%	2.5%	7.1%	5.4%	29.6%	3.5%	10.7%	5.7%
> €40 000 & < €55 000	47.9%	2.9%	7.1%	5.7%	28.5%	3.5%	10.9%	5.9%
≥55 000	47.3%	2.7%	7.0%	5.5%	28.8%	3.5%	10.7%	6.0%
Chronic disease								
No	47.1%	2.8%	7.0%	5.5%	29.3%	3.7%	10.5%	5.9%
One	48.1%	2.4%	7.1%	5.5%	29.0%	3.3%	10.9%	6.0%
Multiple	46.4%	2.9%	7.4%	5.7%	27.7%	3.4%	11.0%	5.8%

Supplementary table S2. Sensitivity analyses with a bandwidth of 6 months and a quadratic polynomial. Regression discontinuity estimates of working months and months in different exit pathways from paid employment comparing the intervention group to the control group as reference[a]

	Addition months spent in paid employment		Working months lost due to early exit through[f]:							
			**Early retirement**		**Disability benefits**		**Unemployment**		**Economic inactivity**	
	Months	95% CI	Months	95% CI	Months	95% CI	Months	95% CI	Months	95% CI
All[b]	**4.19**	**3.15; 5.21**	**-6.62**	**-7.59; 5.64**	**0.49**	**0.08; 0.89**	0.62	-0.01; 1.25	0.51	-0.02; 1.04
Gender[c]										
Female	**1.85**	**0.21; 3.49**	**-5.08**	**-6.56; -3.60**	0.50	-0.13;1.13	**1.09**	**0.16; 2.00**	0.73	-0.41; 1.86
Male	**5.72**	**4.41; 7.03**	**-7.62**	**-8.89; 6.34**	0.43	-0.10; 0.96	0.26	-0.60; 1.12	**0.44**	**0.02; 0.86**
Income[d]										
≤ €25 000	**4.88**	**3.70; 6.06**	**-6.70**	**-6.06; 3.70**	**0.55**	**0.09; 1.01**	**0.76**	**0.04; 1.49**	**0.63**	**0.25; 1.01**
> €25 000 & ≤40 000	**5.17**	**3.05; 7.29**	**-8.30**	**-10.36; 6.26**	**1.18**	**0.25; 2.11**	0.59	-0.81; 1.96	**1.03**	**0.33; 1.73**
> €40 000 & < €55 000	**3.77**	**1.67; 5.88**	**-6.06**	**-8.12; 3.99**	0.79	-0.08; 1.66	0.87	-0.43; 2.17	0.38	-0.21; 0.96
≥55 000	**5.57**	**3.68; 7.46**	**-7.32**	**-9.11; 5.52**	-0.27	-0.87; 0.33	0.92	-0.19; 2.02	0.51	-0.17; 1.18
Chronic disease[e]										
No	**4.47**	**3.01; 5.92**	**-6.02**	**-7.38; -4.66**	0.11	-0.37; 0.59	-0.22	-1.11; 0.67	**0.79**	**0.08; 1.49**
One	**3.38**	**1.56; 5.20**	**-6.69**	**-8.42; -4.96**	**0.79**	**0.04; 1.53**	**1.72**	**0.58; 2.86**	0.16	-0.08; 1.12
Multiple	**4.80**	**2.37; 7.23**	**-8.21**	**-10.48; -5.94**	1.03	-0.23; 2.28	**1.07**	**0.41; 2.56**	0.41	-0.95; 1.76

Significant results (p-value < 0.05) are presented in bold; [a] RD robust design with a bandwidth of 6 months (h=6) and a quadratic polynomial (p=2); [b] Models were corrected for gender, personal gross income and chronic disease; [c] Models were corrected for personal gross income and chronic disease; [d] Models were corrected for gender and chronic disease; [e] Models were corrected for gender and personal gross income; [f] The sum of time in early retirement does not necessarily equal the time spent in other activities because each model is estimated independently and we have not included death as one of the pathways.

Supplementary table S3. Sensitivity analyses with a bandwidth of 3 months and a cubical polynomial. Regression discontinuity estimates of working months and months in different exit pathways from paid employment comparing the intervention group to the control group as reference[a]

	Addition months spent in paid employment		Working months lost due to early exit through[f]:							
			**Early retirement**		**Disability benefits**		**Unemployment**		**Economic inactivity**	
	Months	95% CI	Months	95% CI	Months	95% CI	Months	95% CI	Months	95% CI
All[b]	**2.79**	**0.35; 5.22**	**-6.30**	**-8.66; -3.95**	**0.47**	**0.13; 0.82**	**1.07**	**-0.41; 2.55**	1.19	-0.01; 2.38
Gender[c]										
Female	-2.07	-5.84; 1.71	**-3.94**	**-7.47; -0.41**	-0.37	-1.82; 1.09	**2.67**	**0.66; 4.69**	**3.28**	**0.75; 5.81**
Male	**6.31**	**3.17; 9.45**	**-7.93**	**-11.04; -4.82**	0.33	-0.96; 1.62	-0.21	-2.29; 1.87	-0.35	-1.28; 0.58
Income[d]										
≤ €25 000	-0.86	-5.71; 3.99	**-4.69**	**- 9.00; -0.37**	-0.49	-2.30; 1.33	1.32	-1.57; 4.20	3.48	-0.28; 7.23
> €25 000 & ≤40 000	**5.93**	**0.96; 10.90**	**-10.71**	**-15.73; -5.75**	0.96	-1.31; 3.24	1.21	-1.96; 4.68	**0.62**	**0.01; 1.22**
> €40 000 & < €55 000	1.20	-3.91; 6.31	-3.49	- 8.55; 1.57	0.07	-2.18; 2.32	0.03	-3.16; 3.22	-0.22	-1.56; 1.12
≥55 000	**5.09**	**0.61; 9.56**	**-6.16**	**-10.44; -1.89**	-0.04	-1.42; 1.34	1.53	-1.13; 4.19	-0.40	-1.76; 0.96
Chronic disease[e]										
No	**4.00**	**0.54; 7.46**	**-6.39**	**-9.69; -3.08**	-0.67	-1.85; 0.52	0.48	-1.62; 2.58	-1.41	-0.14; 2.96
One	1.17	-3.13; 5.47	**-5.23**	**-9.41; -1.05**	-0.05	-1.81; 1.71	1.50	-1.20; 4.21	1.09	-1.12; 3.30
Multiple	2.32	-3.28; 7.92	-7.39	-7.92; 3.28	2.73	-0.18; 5.63	1.83	-1.47; 5.13	0.09	-3.04; 3.22

Significant results (p-value < 0.05) are presented in bold; [a] RD robust design with a bandwidth of 3 months (h=3) and a cubical polynomial (p=3); [b] Models were corrected for gender, personal gross income and chronic disease; [c] Models were corrected for personal gross income and chronic disease; [d] Models were corrected for gender and chronic disease; [e] Models were corrected for gender and personal gross income; [f] The sum of time in early retirement does not necessarily equal the time spent in other activities because each model is estimated independently and we have not included death as one of the pathways.

Supplementary table S4. Sensitivity analyses with a bandwidth of 12 months and a cubical polynomial. Regression discontinuity estimates of working months and months in different exit pathways from paid employment comparing the intervention group to the control group as reference[a]

	Addition months spent in paid employment		Working months lost due to early exit through[f]:							
			**Early retirement**		**Disability benefits**		**Unemployment**		**Economic inactivity**	
	Months	95% CI	Months	95% CI	Months	95% CI	Months	95% CI	Months	95% CI
All[b]	**4.84**	**3.54; 6.14**	**-6.33**	**-7.16; -5.50**	**0.47**	**0.13; 0.82**	**0.59**	**0.04; 1.13**	0.39	-0.06; 0.85
Gender[c]										
Female	**1.73**	**0.32; 3.15**	**-4.70**	**-5.97; -3.43**	0.49	-0.05; 1.03	**0.91**	**0.11; 1.72**	0.60	-0.38; 1.59
Male	**5.56**	**4.43; 6.68**	**-7.47**	**-8.56; -6.39**	0.43	-0.03; 0.88	0.36	**-0.37; 1.09**	**0.45**	**0.09; 0.81**
Income[d]										
≤ €25 000	1.52	-0.29; 3.34	**-4.18**	**-5.70; -2.65**	0.21	-0.50; 0.92	0.20	-0.91; 1.31	0.50	0.94; 1.93
> €25 000 & ≤40 000	**5.44**	**3.63; 7.26**	**-8.23**	**-9.98; -6.48**	**0.96**	**0.14; 1.77**	0.73	-0.45; 1.91	0.62	-0.01; 1.22
> €40 000 & < €55 000	**3.77**	**1.98; 5.57**	**-6.20**	**-7.96; -4.44**	**0.91**	**0.17; 1.65**	0.75	-0.35; 1.86	0.38	-0.13; 0.88
≥55 000	**5.37**	**3.75; 7.00**	**-6.85**	**-8.38; -5.31**	-0.15	-0.66; 0.37	0.80	-0.16; 1.76	0.48	-0.11; 1.07
Chronic disease[e]										
No	**4.34**	**3.09; 5.58**	**-6.03**	**-7.19; -4.87**	0.20	-0.21; 0.60	-0.09	-0.86; 0.67	**0.68**	**0.07; 1.29**
One	**3.19**	**1.63; 4.75**	**-6.43**	**-7.90; -4.96**	0.73	**0.09; 1.38**	**1.65**	**0.67; 2.63**	0.28	-0.56;1.11
Multiple	**4.66**	**2.56; 6.77**	**-7.23**	**-9.19; -5.26**	0.71	-0.39; 1.81	0.67	-0.61; 1.96	0.05	-1.09; 1.18

Significant results (p-value < 0.05) are presented in bold; [a] RD robust design with a bandwidth of 12 months (h=12) and a cubical polynomial (p=3); [b] Models were corrected for gender, personal gross income and chronic disease; [c] Models were corrected for personal gross income and chronic disease; [d] Models were corrected for gender and chronic disease; [e] Models were corrected for gender and personal gross income; [f] The sum of time in early retirement does not necessarily equal the time spent in other activities because each model is estimated independently and we have not included death as one of the pathways.
